# Risk Screening Tools Could Potentially Miss HIV-Positive Individuals Who Seek Testing Services: A Secondary Program Data Analysis on the Performance Characteristics of an Adolescent and Adult HIV Risk Screening Tool in Uganda

**DOI:** 10.3390/tropicalmed9020037

**Published:** 2024-02-01

**Authors:** Marvin Lubega, Katherine Guerra, Megan Ginivan, Yewande Kamuntu, George Senyama, Andrew Musoke, Fiona Gambanga, Shaukat Khan, Geoffrey Taasi, Sylivia Nalubega, John Bosco Junior Matovu

**Affiliations:** 1 Clinton Health Access Initiative-Uganda, Moyo Close, Kampala P.O. Box 2191, Uganda; mlubega@clintonhealthaccess.org (M.L.); kguerra@clintonhealthaccess.org (K.G.); mginivan@clintonhealthaccess.org (M.G.); ykamuntu@clintonhealthaccess.org (Y.K.); gsenyama@gmail.com (G.S.); amusoke@seedglobalhealth.org (A.M.); fgambanga@clintonhealthaccess.org (F.G.); sakhan@gmail.com (S.K.); 2 AIDS Control Program, Ministry of Health, Plot 6, Lourdel Road, Kampala P.O. Box 7272, Uganda; taasi.taasi@gmail.com; 3 Institute of Applied Technology, Fatima College of Health Sciences, Ajman P.O. Box 3798, United Arab Emirates; syliviaogwang@yahoo.com; 4 ICAP at Columbia University, Nairobi P.O. Box 29840-00202, Kenya

**Keywords:** HIV testing services, HIV risk screening, HIV risk screening tools, misclassification, HIV testing cost analysis

## Abstract

Improving HIV testing efficiency saves financial and material resources for health. We conducted a secondary data analysis of routinely collected HIV risk-screening program data in Uganda, from October to November 2019, to determine the performance characteristics of the adolescent and adult HIV risk screening tools in public health facilities. A total of 19,854 clients had been screened for HIV testing eligibility and tested for HIV. The overall positivity rate (cluster-weighted prevalence of HIV) among those screened was 4.5% (95% CI: 4.1–4.8) versus 3.71% (95% CI: 3.06–4.50) among those not screened. The sensitivity and specificity of the risk screening tool were 91% (95% CI: 89–93) and 25% (24.2–26), respectively. With screening, the number needed to test to identify one PLHIV was reduced from 27 to 22. Although risk screening would have led to a 24.5% (4825/19,704) reduction in testing volume, 9.3% (68/732) of PLHIV would have been missed and be misclassified as not eligible for testing. The cost saving per PLHIV identified was minimally reduced by 3% from USD 69 without screening to USD 66.9 with screening. Since the treatment-adjusted prevalence of HIV is dropping globally, overzealous use of risk screening tools to determine who to test or not carries the potential of missing PLHIV due to their limited specificity. We recommend the use of scientifically validated HIV risk screening tools, and a need to explore the use of HIV self-testing as a test for tirage to minimize misclassification of people who seek HIV testing services.

## 1. Introduction

Globally, human immune-deficiency virus (HIV) testing programs have contributed to enormous progress in identifying people living with HIV (PLHIV) and increased coverage of anti-retroviral therapy (ART) [1]. By 2020, Uganda was nearing the first of the United Nation’s 95–95–95 targets of identifying 95% of PLHIV with approximately 91% of the 1.4 million PLHIV already identified, and 94% on treatment but with a slightly lower viral suppression rate (85%) [2]. Despite remarkable progress in HIV case identification in Uganda, there were approximately 126,000 PLHIV not yet on treatment with an estimated 38,000 new HIV infections by the year 2020, indicating a need to sustain innovative HIV case identification approaches and linkage to treatment.

Uganda and many other countries in sub-Saharan Africa (where the HIV burden is greatest in the world) are about to attain the first of the three 95% targets (95% of PLHIV know their HIV status) and identifying the remaining undiagnosed PLHIV is becoming increasingly difficult and resource intensive. At the same time, resources for HIV testing services (HTS) are declining; hence, countries are under pressure to maximize testing efficiency by focusing on increasing HIV testing yield at both community and facility-based testing entry points [3]. In 2017, for example, the President’s Emergency Plan for AIDS Relief (PEPFAR) had a target of conducting approximately 7.2 million HIV tests in Uganda, but this target dropped to just over 1.7 million in 2020, with targets for provider-initiated testing and counseling (PITC) reduced and replaced by “high-yield” testing approaches such as index testing [4].

Uganda employs a strategic model mix of HIV testing modalities and approaches throughout the country, including facility and community-based HTS models. Since the introduction of more targeted HIV testing approaches such as partner testing services, the overall HTS yield remained relatively constant from 3.1% in 2017, peaking at 3.8% in 2018 and regressing to 3.1% in 2019. Whereas index testing (including partner testing services) provided a positivity rate of 20% in 2019, the overall contribution to case identification by this HTS modality was only 15.4%, with provider-initiated counseling and testing other (PITC-other) modalities contributing 53% of all HIV-positive cases identified [5]. Large facility-based entry points such as the outpatient departments (OPD), however, reach large numbers of clients and hence PITC modality at OPD can identify more PLHIV in absolute numbers than high-yield targeted strategies. As a result, in recent years, the vast majority of newly identified PLHIV have been identified through facility-based testing [6]. For example, in 2018, PITC at OPD, and facility-based voluntary counseling and testing (VCT) accounted for approximately 68% of all newly diagnosed PLHIV [7].

Uganda traditionally offers the opt-out testing approach where a person is notified that HIV testing is part of normal care for everyone, but they can decline a test. This approach provides an opportunity for every person who visits a health facility, whether sick or not, to know their HIV status. Given resource constraints, ministries of health are looking for opportunities to improve HIV testing efficiency by reducing the total number of people tested for HIV while targeting to identify the same or higher numbers of PLHIV. To achieve this, many countries have institutionalized HIV risk screening tools, which consist of a combination of clinical and behavior-based criteria, used to identify individuals with high risk of HIV infection who are then prioritized for testing. Screening tools have been used successfully for pediatric and adolescent clients to identify children/adolescents living with HIV and are used to screen in children and adolescents for HIV testing using socio-demographic and clinical variables [8]. However, evidence around the use of screening tools in adults is limited [9]. Evaluations of behavior-based risk algorithms in the United States and Malawi to identify sexually transmitted infection (STI) indicate that screening tools have varying sensitivities and specificities [10], and, hence, carry a potential to screen out people (misclassification). The underlying hypothesis is to reduce the total number of tests while increasing the positivity rate.

The HTS program at the Ministry of Health (MOH) in Uganda introduced an HIV risk screening tool to determine eligibility for HIV testing among clients attending OPD clinics in 2019. Before its deployment, the tool had been field tested at two high-volume urban health facilities, where 500 people were screened and tested for HIV upon consent through routine provider-initiated HIV counseling and testing. The field-tested tool exhibited a sensitivity of 85% and a specificity of 62%, but there were no records of predictive values in the field-testing report. The tool was modified, and screening questions were reduced from thirteen to seven because some clients felt uncomfortable responding to these questions, while some questions were subsets of others, so they were merged. In October 2019, MOH deployed this tool for use in 24 public health facilities, targeting clients aged 15 years and above in both OPD and VCT departments.

The screening tool included the following questions:

1. Does the patient have co-morbidities or an exposure risk?
  - Presumptive TB;
  - New perpetuators and survivors of SGBV;
  - A reactive HIV self-test;
  - Elicited through index testing;
  - Accidentally exposed to HIV;
  - Diagnostic HTS (unconscious, critically ill, mentally impaired).
2. Has the client had an HIV test in the last 12 months?
3. Has the client tested within the last 3 months?
4. Has the client had unprotected sex with a partner(s) of unknown HIV status or known HIV-positive status since the last negative HIV test?
5. HIV-negative partner(s) in a discordant relationship and has not had an HIV test within the past 3 months.
6. The client has a diagnosis of sexually transmitted infection (including Hepatitis B) after a previous negative HIV test.
7. A client with TB, STI, or Hepatitis B, is symptomatic of HIV, or is on PEP and tested HIV-negative at least 1 month ago.

A client was eligible for HIV testing if any of the responses were “Yes” and ineligible for testing if all the responses were “No”.

We aimed to determine the performance of the screening tool in public health facility settings by assessing the diagnostic characteristics of the tool in terms of sensitivity, specificity, predictive values and number needed to test to identify an individual with HIV (primary outcome). We also aimed to determine the cost implication of using or not using the screening tool by analyzing the estimated comparative costs of testing with and without screening (secondary outcome).

## 2. Materials and Methods

### 2.1. Design

We conducted a secondary data analysis of de-identified and anonymized HIV testing program data, collected and reported by 24 public health facilities in Uganda. The de-identified data was requested from the MOH for secondary analysis and upon approval, was extracted from the DHIS2 by district biostatisticians. Data analysis was performed between March and April 2020 on all HIV risk screening data collected and reported between October and November 2019. The primary outcome was to compare the sensitivity and specificity of the screening tool with the standard of care (testing without screening), and the secondary outcome was to estimate the cost savings of using the screening tool compared to the standard of care.

### 2.2. Settings

The adolescent and adult HIV risk screening tool was deployed at the OPD clinics in 24 health facilities commencing October 2019. The use of the screening tool was integrated into routine care, and all clients who sought HTS in the OPD clinics consented and were screened by healthcare providers to determine eligibility for HIV testing. Because clients who attended HIV testing points in the OPD and VCT clinics had turned up for HIV testing, the use of the screening tool did not exclude anyone from testing; hence, whoever was categorized as either eligible or ineligible for testing upon screening was offered HIV testing services, including testing and linkage to posttest services. In line with national guidelines, the screening tool was only administered to clients over the age of 15 years. Screening and testing information was recorded using the national health management information system using primary data collection registers. We analyzed data that had been decoded, anonymized, and reported by the health facilities in the district health information system (DHIS2).

### 2.3. Study Participants

The data analysis included entries of all clients aged 15 years and above who were screened for HIV testing eligibility and tested at the 24 health facilities in the months of October and November 2019. All clients who sought HTS at the 24 health facilities were screened for eligibility as a routine service, and, irrespective of the eligibility outcome (eligible, not eligible), all were offered non-coercive (voluntary) HIV testing.

### 2.4. Study Variables

The predictor for the primary outcome was binary, “eligible for screening”, or “not eligible for screening” and the outcome variable was the HIV test result “Tested HIV Positive”, or “Tested HIV Negative”. Data on these outcomes was collected for all clients who had been screened and tested irrespective of the screening eligibility (whether they were eligible or not eligible for testing). The HIV testing outcomes were stratified by social demographic characteristics including age, gender, marital status and eligibility screening outcome.

### 2.5. Sample Size and Data Sources

We included all (census) data submitted into the DHIS2 for clients screened for HIV eligibility at 24 public health facilities in the two-month period (October and November 2019). The data source was DHIS2 (secondary data) from districts where the health facilities belonged. By considering all data submitted from all facilities, we excluded selection bias which would result from using a small sample. The choice of the 24 health facilities was made because these were the first facilities to use the screening tool and data from these facilities would inform further scale-up of risk screening to determine HIV testing eligibility.

### 2.6. Data Analysis

Data were abstracted from the DHIS2 by district biostatisticians and shared with the authors for analysis. The data were checked for consistency and accuracy, cleaned using Excel software (Microsoft 365) and exported into STATE/SE 15 (StataCorp, College Station, TX, USA) software for analysis. We included a record that reported both the screening eligibility and a documented HIV test result. We excluded entries with missing screening eligibility, missing HIV test results and those reporting an age below 15 years. HIV positivity rates with and without screening were calculated allowing for clustering by facility. To obtain the combined proportion across facilities, we aggregated the proportions calculated within each cluster from the cluster analysis conducted using Stata. This involved summing up the proportions across all clusters and deriving an overall proportion that represents the combined results from the facilities in our study. We computed the sensitivity, specificity, predictive values and number needed to test (NNT) to identify one HIV-positive person with or without screening.

Sensitivity (true positive rate) was defined as the probability that a client was screened eligible for testing and tested HIV positive, and was calculated as the proportion of individuals who tested HIV positive among those who were screened eligible for testing. Specificity (true negative rate) was defined as the probability that a client was screened ineligible for testing and tested HIV negative and was calculated as the proportion of individuals who tested HIV negative among those who were screened ineligible for testing. Positive Predictive Value (PPV) was defined as the proportion of clients who were eligible for testing and tested HIV positive out of all those who screened eligible. Negative Predictive Value (NPV) was defined as the proportion of clients who were screened ineligible for HIV testing and tested HIV negative, out of all those who were screened ineligible. Sensitivity analysis was performed for every screening question by computing the proportion of individuals who tested HIV positive among those who were screened as eligible for testing by that screening question. Logistic regression with random effects was used to estimate the odds ratio (OR) associated with HIV status for each variable at a 95% confidence level across social and demographic characteristics, including eligibility for testing as a predictor for HIV status.

For the secondary outcome, we estimated the unit cost needed to identify one HIV-positive client with or without risk screening from which we determined the cost difference. Cost estimates (in USD) were calculated based on commodity and human resource (HR) required to conduct HIV testing for clients who were categorized as eligible. Commodity costs were based on the public procurement cost of HIV rapid diagnostic tests in Uganda at the time of screening. Human resource costs were calculated using the average salary for counselors in public health facilities. Time requirements for the standard of care (no screening in HTS) assumed the time for counseling and testing as was outlined in national guidelines while screening time assumptions were based on implementing partners’ (IPs) reports. Estimated costs did not include operational costs, such as training and the printing of risk screening tools.

### 2.7. Ethical Considerations

The authors analyzed anonymized secondary program data which did not include client identification information. Neither the authors nor the district biostatisticians interacted with any primary client records before, during, or after the data analysis. The analyzed data had been decoded at the time of reporting into the DHIS2; hence, access to this data did not pose a breach of confidentiality or would not potentially lead to the identification of clients. For this reason, the authors did not seek Institutional Review Board approval but sought clearance from the Ministry of Health to access the secondary data from district DHIS2 systems through district biostatisticians.

## 3. Results

**Figure 1:**
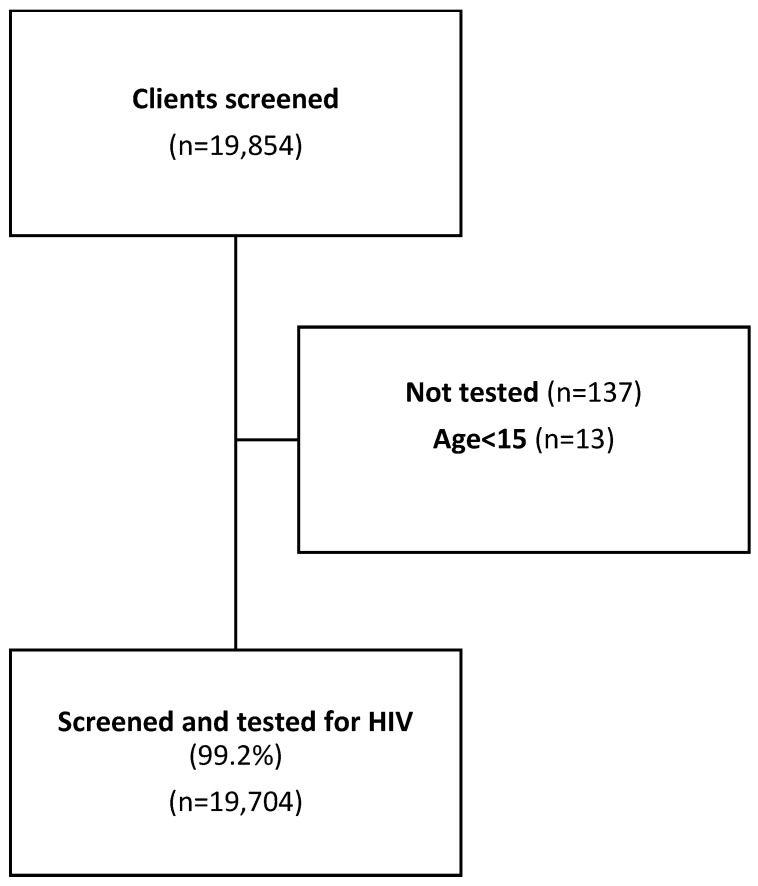
Study flow chart.

A total of 19,854 clients were screened for HIV testing eligibility; we excluded 137 records due to missing HIV testing information, and an additional 13 records were excluded because the documented age was below 15 years (Figure 1).

Of the remaining 19,704 (99.2%) clients, 12,971 (66%) were female, with a median age of 27 years (IQR: 21–35) (Table 1).

The overall positivity rate (cluster-weighted prevalence of HIV) was estimated at 3.71% (95% CI: 3.06–4.50), which would be the yield without screening. Among those screened, the positivity rate was 4.5% (95% CI: 4.1–4.8). The sensitivity of the tool was 91% (95% CI: 88–93) while the specificity was 25% (24–26). Of the 68 people who were screened out, 50 were females aged 16 to 52 years and 33 were married. The 18 male clients were aged 24 to 47 years and 11 were married. Without screening, at a positivity rate of 3.7%, the number of people that needed to be tested to identify one HIV-positive individual was 27 (1/3.7 × 100). With screening at a positivity rate of 4.5%, the number of people that needed to be tested to identify one HIV-positive individual reduced to 22 (1/4.5 × 100) (Table 2).

The positive likelihood ratio, which was defined as the probability that a client was screened eligible and tested HIV positive divided by the probability a client was screened eligible and tested HIV negative was 1.2, meaning that clients who were screened and found to be eligible for HIV testing were 1.2 times more likely to test HIV positive on the national HIV testing algorithm (standard of care). The negative likelihood ratio, which is the probability that a client was screened ineligible but tested HIV positive, divided by the probability a client was screened ineligible and tested HIV negative, was 0.4, meaning that ineligible clients were less likely to test HIV positive on confirmation by 0.4 times. Overall, screening for HIV testing eligibility using the screening tool would have led to a 24.5% (4825/19,704) reduction in testing volume but 9.3% (68/732) of PLHIV would be missed having been misclassified as not eligible for testing. The screening tool questions had varied sensitivity, as presented in Table 3 below.

Not having tested in the last 12 months was the most sensitive screening question (42.9%) while the client having a diagnosis of sexually transmitted infection was least sensitive (2.9%) and would miss up to 97% of PLHIV.

The cost per PLHIV identified was slightly reduced by 3%, from USD 69 without screening to USD 66.9 with the implementation of the screening tool (Table 4).

## 4. Discussion

Over the past few years, there has been a strong narrative supporting the use of risk screening tools to improve testing efficiency with very limited evidence of their impact. This program data analysis identifies operational gaps in HIV case identification among clients who seek health services at outpatient departments and highlights how HIV risk screening tools may misclassify HIV-positive clients as “not at risk” of being HIV positive. These findings relate to those of Antelman and colleagues [11], who reported that a risk screening tool for children and adolescents in Tanzania missed an unacceptably high proportion (36%) of HIV-positive children. Such missed opportunities may propagate HIV transmission resulting from being unaware of the positive HIV status and may lead to delayed diagnosis and linkage to treatment resulting in AIDS-related deaths.

This study and research from other countries [12] show that screening tools could reduce testing volumes by 24%; hence, apparently saving the cost per HIV-positive case identified, screening in the current study resulted in a marginal increase in positivity rate from 3.71% to 4.5%. Of more concern is the number of clients who were misclassified as ineligible for HIV testing, even though they were HIV positive and they were willing to test for HIV. Opting to test for HIV means they had a perceived risk of being HIV positive and would not need to undergo another layer of screening; thus, screening would deny them a chance to know their HIV status. This finding is supported by observations of a systematic review and metanalysis [13] on the uptake of three HIV testing approaches, i.e., opt-in, opt-out and risk-based testing, which recommended the opt-out approach as the best, followed by the opt-in approach. While the crude sensitivity of the tool was 91%, individual questions exhibited varying sensitivities, ranging from 2.9% (client has a diagnosis of a sexually transmitted infection (including Hepatitis B) after a previous negative HIV test) to 42.9% (not having tested in the last 12 months). Since a “yes” to any of the screening questions would render the client eligible for HIV testing, it means more HIV-positive clients can be identified by using a tool that has several independent risk questions, and this would minimize the potential to miss PLHIV.

HIV risk screening tools with low specificity can misclassify HIV-positive clients and render them ineligible for HIV testing, and this can retard the attainment of HIV case identification targets even though the target of testing fewer people could be achieved. For example, in 2021, Uganda conducted approximately 4,608,652 HIV tests, of which 1,753,704 (38%) were in OPD. Applying a screening tool with a sensitivity of 91% at an average national positivity rate of 2.85% would result in missing about 4500 PLHIV; hence, it is essential for HTS programs to weigh this impact as they seek to implement the use of risk screening tools. Moreover, the Uganda national DHIS2 data (2018–2020) show a consistent decline in the total number of PLHIV identified as the number of people tested for HIV reduced. In 2018, 8,473,627 were tested versus 280,767 identified; in 2019, 6,384,317 were tested versus 199,743 identified; and in 2020, 6,355,186 were tested versus 164,804 identified. Whereas the country has not performed statistical analysis to correlate the reduction in HIV testing volumes with the number of HIV-positive clients that could be potentially missed, our data analysis, plus the published literature suggests the potential of missing HIV-positive people through the use of risk screen-out tools [14].

Global guidance exists on the optimal diagnostic characteristics of a risk screening tool and the literature quotes a sensitivity of 90% and above as acceptable for a risk-based screening tool [12]. With a declining HIV prevalence (treatment-adjusted prevalence), a sensitivity of 95% and above is desirable and acceptable if Uganda and other countries are to attain the first 95 of the global targets. Alternative HIV case-finding approaches have been suggested to enhance the identification of PLHIV who could be missed by risk screening tools, and these include index testing, social network testing, use of HIV self-testing and regular retesting of people with ongoing risk of acquiring HIV [15]. Of these, HIV self-testing would be the ideal option for people who are categorized as ineligible for testing because being an antibody-based test, it is more sensitive and specific than risk-based screening tools. In their paper entitled, “The future of HIV testing in Eastern and Southern Africa: Broader scope, targeted services”, Anna Grimsrud et al. recommend a shift away from yield/positivity and case identification as the sole or primary indicators of HTS program success, and instead focus on maximizing the absolute number of HIV diagnoses [14] and linking high-risk individuals to combination prevention services to minimize their chances of acquiring HIV. This contributes to the status-neutral HIV testing approach where PLHIV have undetectable viral loads and negligible chances of transmitting HIV, while the high-risk negative have negligible chances of acquiring HIV.

From a cost perspective, risk screening slightly reduced the cost per PLHIV identified by about 3% resulting in overall program savings from the lower commodity requirements for reduced testing volumes. This finding is comparable to that of a Zimbabwean validation study for an adolescent screening tool in which they found the tool to be cost saving in a community setting and recommended it for use in low-resource settings [8]. However, in our study, these savings only accounted for human resource and commodity costs and do not reflect the full costs of implementing screening tools, which would include training, printing, dissemination of tools, monitoring and evaluation, among others. In addition, these savings were at the expense of missed PLHIV and would be offset by the cost to reach these missed PLHIV through alternative strategies such as index testing.

Facility-based testing is generally less expensive compared to community testing strategies such as outreach and mobile testing; hence, all opportunities need to be maximized to identify HIV-positive clients who visit health facilities. If clients living with HIV are screened out at facilities, ministries need to consider if they will ultimately identify these clients through alternative, more expensive HIV testing approaches. Moreover, it is of ethical concern that HIV-positive clients are misclassified and denied HIV testing. Ong and colleagues [16] share similar views and caution against the urge to use screening tools to minimize costs, risking missing out on HIV-positive people. These researchers recommend instead the adoption of scientifically validated screening tools in settings where routine HIV testing may be difficult to achieve, a recommendation also made by other researchers [9].

Routine use of risk screening tools would require training and supervision of HIV testers to minimize user errors that lead to misclassification of clients. Supportive supervision reports by Uganda’s Ministry of Health indicated that some healthcare workers did not follow the screening standard operating procedure leading to misclassification of clients. Although training and ongoing mentorship would improve healthcare workers’ capacity to screen, this would probably only address the misclassification bias to a limited extent; for example, if clients do not feel comfortable answering the screening questions truthfully, the risk screening tool would not detect the misclassification. Improving the sensitivity of the tool would require the formulation of less stigmatizing questions and calls for the allocation of more private spaces for responses, as cited by WHO in Quinn and Wong [15]. This is an area for further study to determine the extent to which misclassification bias can be reduced.

The costs and implications of failing to identify PLHIV within health facilities where they could be identified and linked to care may outweigh savings in testing commodities and calls for strategic reforms by countries to consider alternatives to risk screening tools. Recently, there has been growing advocacy for countries to adopt HIV self-testing (HIVST) as a screening approach (test for triage) for clients seeking HIV testing at both facility and community levels. Scaling up HIVST would require the formulation or adoption of HIVST policies and a considerable financial investment to roll out these policies, including commodity management. Recent research in Malawi has shown the potential of HIVST to expand testing coverage, while reducing human resource time and limiting the risk of screening PLHIV out, given the much higher sensitivity of antibody screening platforms compared to risk-based screening tools [17].

Our results show that the risk screening tool helped to reduce the testing volumes and improved HIV testing efficiency but at the same time misclassified people who were HIV positive. Much as countries are exploring the use of HIV risk screening tools to identify PLHIV more efficiently and make better use of available resources, the evidence presented above clearly illustrates the tradeoffs involved in implementing these tools. Since the majority of PLHIV have been identified globally, and, narrowing this to individual countries, the reliance on risk screening tools to classify who is likely to be HIV positive may be counterproductive, especially in low HIV prevalence countries or in high prevalence countries but with a low HIV treatment adjusted prevalence. For such countries, an antibody/antigen screening test would be ideal.

Limitations: This was a secondary analysis of routine program data from 24 health facilities that were not randomly selected and may not represent the over 3000 health facilities that offer HTS in the country. Costing included commodity and human resource costs, as these are primary cost drivers for HTS, but was not exhaustive and did not include operational costs. Human resource time requirements were estimated based on guidelines and implementing partner reports, rather than time-in-motion studies.

## 5. Conclusions

The use of the HIV risk screening tool in OPD settings in Uganda demonstrated improved HIV testing efficiency by reducing testing volumes but resulted in screening out 9 out of every 100 people living with HIV due to its low specificity. There were minimal cost savings earned through testing fewer people because of screening, but these would be offset by the cost to reach the missed PLHIV through alternative and more expensive HIV testing strategies, such as index testing.

Recommendations: The team recommends the use of scientifically validated HIV risk screening tools by countries; ministries should provide regular support, supervision and mentorship to all HIV testers to ensure adherence to the risk screening SOPs. To limit the misclassification of clients seeking HTS, the use of facility-based HIVST (HIV antibody test) should be explored. Scientific validation of the risk screening tool using a statistically representative sample is recommended to generate generalizable results.

## Figures and Tables

**Table 1:** Baseline characteristics stratified by HIV status.

Characteristics	All	HIV Status		Univariate OR (95% CI)	*p* Value
		Positive	Negative		
	N = 19,704	n = 732 (%_col_)	n = 18,972 (%_col_)		
**Age (years)**					
15–19	3379 (17%)	42 (6%)	3337 (17%)	1	<0.001
20–35	11,469 (58%)	457 (62%)	11,012 (58%)	3.10 (2.08–4.63)	
36–50	3310 (17%)	171 (24%)	3139 (17%)	4.21 (2.78–6.35)	
50+	1546 (8%)	62 (9%)	1484 (8%)	3.33 (2.00–5.56)	
**Median Age (IQR)**	27 (21–35)	30 (25–39)	26 (21–35)		
**Gender**					
Female	12,971 (66%)	475 (65%)	12,496 (66%)	1	0.7762
Male	6733 (34%)	257 (35%)	6476 (34%)	1.01 (0.83–1.24)	
**Marital Status**					
Married	12,071 (61%)	464 (63%)	11,607 (61%)	1	<0.001
Divorce/Separated	1544 (8%)	115 (16%)	1429 (8%)	2.11 (1.59–2.81)	
Single	5628 (29%)	128 (18%)	5500 (29%)	0.60 (0.43–0.85)	
Widowed	461 (2%)	25 (3%)	436 (2%)	1.60 (0.88–2.88)	
**Screened and Eligible for Testing**					
Yes	14,879 (76%)	664 (91%)	14,215 (75%)	3.60 (2.30–5.62)	
No	4825 (24%)	68 (9%)	4757 (25%)	1	<0.001

**Table 2:** Diagnostic characteristics of the adult HIV risk screening tool.

Variable	Tested (N)	Positive (n)	Positivity Rate (n/N%, 95% CI)
**Positivity Rate**			
Without risk screening	19,704	732	3.7%
With risk screening (screened in)	14,879	664	4.5%
With risk screening (screened out)	4825	68	1.4%
**Diagnostic Characteristics of The tool**	(N)	(n)	(n/N%, 95% CI)
Sensitivity	732	68	91%, (89–93)
Specificity	19,636	14,879	25%, (24–26)
Positive Predictive Value			4.5%, (4.2–4.8)
Negative Predictive Value			98.6% (98.3–98.9)
**Number Needed to Test**			
Number needed to test without screening	3.7	1	27
Number needed to test with screening	4.5	1	22
**Likelihood Ratios**			
Positive Likelihood Ratio		1.2	
Negative Likelihood Ratio		0.4	

**Table 3:** Sensitivity analysis for each of the screening questions.

	Eligible (%)	HIV Positivity (%)	Sensitivity	PLHIV Missed (%)
			(95% CI)	
**No HIV test in the last 12 months**	6880 (35%)	314 (4.6%)	42.9% (39.3–46.6)	418 (57.1%)
Patient belongs to 1 of 6 categories in Question 1 of screening tool	936 (5%)	104 (11.1%)	14.2% (11.8–16.9)	628 (85.8%)
Tested in the last 12 months, but not in the last 3 months	4314 (22%)	138 (3.2%)	18.9% (16.1–21.9)	594 (81.1%)
Client has had unprotected sex with partner(s) of unknown HIV status or known HIV-positive status since the last negative HIV test?	3281 (17%)	160 (4.9%)	21.9% (18.9–25.0)	572 (78.1%)
HIV-negative partner(s) in a discordant relationship and has not had an HIV test within the past 3 months	384 (2%)	36 (9.4%)	4.9% (3.5–6.7)	696 (95.1%)
Client has diagnosis of sexually transmitted infection (including Hepatitis B) after previous negative HIV test	528 (3%)	21 (4.0%)	2.9% (1.8–4.4)	711 (97.1%)
Client with TB, STI, Hepatitis B, symptomatic of HIV, or is on PEP and tested HIV negative at least 1 month ago	750 (4%)	44 (5.9%)	6.0% (4.4–8.0)	688 (94%)
Screening tool (yes to any of above questions)	14,885 (76%)	664 (4.46%)	90.7% (88.4–92.7)	68 (9.3%)
No screening tool	19,717	732 (3.71%)		

**Table 4:** Cost analysis (USD) for implementing HIV screening using a risk screening tool at an outpatient department (OPD) in Uganda.

Human Resource and Commodity Costs (USD) for Current Standard of Care Compared with Screening in OPD			
	Standard of Care	Screening in OPD	Screening Tool Savings
Total number of tests (A1)	19,704	16,764	2951
Total cost	USD 44,357	USD 40,222	USD 4138
Commodities	USD 25,825	USD 22,036	USD 3790
Human resources	USD 18,532	USD 18,184	USD 348
Cost per PLHIV identified	USD 69.05	USD 66.91	USD 2.14
Commodity cost per PLHIV identified	USD 40.2	USD 34.4	USD 5.9
Human resources per PLHIV identified	USD 28.85	USD 28.31	USD 0.54

## Data Availability

There is no restriction to access the dataset used to perform the analysis, and the data are available via this link: https://doi.org/10.5061/dryad.m0cfxpp8t (accessed on 23 August 2023).
